# Prognostic Nutritional Index and Oxygen Therapy Requirement Associated With Longer Hospital Length of Stay in Patients With Moderate to Severe COVID-19: Multicenter Prospective Cohort Analyses

**DOI:** 10.3389/fnut.2022.802562

**Published:** 2022-04-05

**Authors:** Alan L. Fernandes, Bruna Z. Reis, Igor H. Murai, Rosa M. R. Pereira

**Affiliations:** ^1^Rheumatology Division, Faculdade de Medicina da Universidade de São Paulo, Hospital das Clinicas HCFMUSP, São Paulo, Brazil; ^2^Department of Nutrition, Center for Health Sciences, Federal University of Rio Grande do Norte, Natal, Brazil

**Keywords:** SARS-CoV-2 infection, COVID-19, malnutrition, prognosis, nutritional status, assessment, prognostic nutritional index (PNI)

## Abstract

**Purpose:**

To evaluate whether the prognostic nutritional index (PNI) is related to the oxygen therapy requirement at hospital admission and to ascertain the prognostic effect of the PNI and the oxygen therapy requirement as predictors of hospital length of stay in patients with moderate to severe coronavirus disease 2019 (COVID-19).

**Methods:**

This is a *post-hoc* analysis in hospitalized patients with moderate to severe COVID-19. The participants were categorized: (1) non-oxygen therapy (moderate COVID-19 not requiring oxygen therapy); (2) nasal cannula therapy (severe COVID-19 requiring nasal cannula oxygen therapy); and (3) high-flow therapy (severe COVID-19 requiring high-flow oxygen therapy). PNI was calculated for each patient according to the following equation: serum albumin [g/dL] × 10 + total lymphocyte count [per mm^3^] × 0.005. The participants were categorized into malnutrition (PNI <40), mild malnutrition (PNI 40–45), and non-malnutrition (PNI > 45).

**Results:**

According to PNI, malnutrition was more prevalent in the high-flow therapy group (94.9%; *P* < 0.001) with significantly lower PNI compared to both groups even after adjusting for the center and C-reactive protein. Patients in the high-flow therapy group [9 days (95% CI 7.2, 10.7), *P* < 0.001] and malnutrition status [7 days (95% CI 6.6, 7.4), *P* = 0.016] showed a significant longer hospital length of stay compared to their counterparts. The multivariable Cox proportional hazard models showed significant associations between both oxygen therapy requirement and PNI categories and hospital discharge.

**Conclusion:**

In addition to oxygen therapy requirement, low PNI was associated with longer hospital length of stay. Our findings suggest that PNI could be useful in the assessment of nutritional status related to the prognosis of patients with moderate to severe COVID-19.

## Introduction

Nutritional assessment emerges as an indispensable management at hospital admission because malnourished patients imply a significantly longer hospital length of stay and high mortality rates (1). Regarding coronavirus disease 2019 (COVID-19), the results have been similar. A retrospective observational study (2) assessing clinical and nutritional characteristics at hospital admission reported a higher risk of in-hospital mortality associated with poor nutritional status. COVID-19, especially severe to critical ones, deals with hyperinflammation as well as immune-cell hyperactivation (3), and a proper nutritional assessment, such as the prognostic nutritional index (PNI), hampers adverse events related to malnutrition (4).

The prognostic nutritional index (PNI) is based on serum albumin concentration and lymphocyte count (5), both usual prognostic markers of the immune system that allow a feasible and objective assessment of nutritional status avoiding poor outcomes (6, 7). This index has been widely adopted as an important tool in patients undergoing renal (8) and cardiac (9)surgery, in acute heart failure (10), in different neoplasms (11, 12), including those undergoing radiotherapy (13), and hospitalized patients with COVID-19 (14–16). In a small-scale cohort study (14), De Lorenzo et al. evaluated the relationship between percentage of fat mass and immune-inflammatory response of 22 adult patients with COVID-19 from baseline to 10th day at the intensive care unit (ICU). The results indicated that obese patients have a compromised immune response, higher inflammation, and reduced PNI, suggesting an influence of fat mass on nutritional status of malnourished patients with COVID-19 admitted to the ICU (14). Corroborating these results, Cinar et al. (15) demonstrated in a retrospective cross-sectional study that lower PNI was independently related to a higher in-hospital mortality in patients with elevated cardiovascular risk factors and COVID-19, who commonly have marked lymphopenia (17) and hypoalbuminemia (18) which potentially reduce PNI and promote the progression of COVID-19 (19).

According to a recent meta-analysis (16), the pooled odds ratios for in-hospital mortality and disease severity were 20 and 22% lower in favor of high PNI values compared to reduced ones, respectively, reaffirming PNI as an independent predictor of in-hospital mortality and disease severity in COVID-19 patients. In addition, cross-sectional data from the aforementioned authors (16) reported lower PNI, higher LDH, and higher D-dimer levels as independent risk indicators of in-hospital mortality. In addition, patients with a history of diabetes mellitus, reduced PNI, and elevated serum levels of lactate dehydrogenase showed a higher tendency to develop severe diseases. However, all studies included in the meta-analysis had a retrospective design and were significantly heterogeneous, which are important limitations to be considered.

So far, there is no data concerning prospective PNI assessment in patients with COVID-19 according to illness severity. Thus, this is the first longitudinal multicenter study evaluating whether nutritional status, assessed by the PNI, is related to oxygen therapy requirement at hospital admission and ascertains the prognostic effect of the PNI and the oxygen therapy requirement on hospital length of stay in patients with moderate to severe COVID-19.

## Materials and Methods

### Study Population

This is a *post-hoc* analysis from a multicenter, double-blind, placebo-controlled, randomized clinical trial (20), and a prospective cohort study (21) conducted in hospitalized patients with moderate to severe COVID-19. Herein, we report a cross-sectional analysis performed on pooled baseline data (at hospital admission) collected from both studies conducted between June 2, 2020 and July 21, 2020 at the Clinical Hospital of the School of Medicine of the University of São Paulo, and July 22, 2020, and September 25, 2020 at the Ibirapuera Field Hospital. In addition, we performed a *post-hoc* analysis from the multicenter prospective cohort study (21) regarding the oxygen therapy requirement and PNI as predictors of hospital length of stay. The screening criteria were the same for both centers, and the end of follow-up occurred on October 7, 2020. All patients had positive COVID-19 diagnosis confirmed by polymerase chain reaction (PCR) testing at study admission or by serology assay (ELISA) to detect IgG against severe acute respiratory syndrome coronavirus 2 (SARS-CoV-2) throughout the study.

An approval was granted by the Ethics Committee of the Clinical Hospital of the School of Medicine of the University of São Paulo (approval numbers: 38237320.3.0000.0068) and the Ethics Committee of the Ibirapuera Field Hospital (approval numbers: 30959620.4.0000.0068). All the procedures were conducted in accordance with the Declaration of Helsinki. Written informed consent was obtained from all individual participants included in the study before being enrolled. The manuscript had critical revisions and important intellectual content and was approved for publication by all the authors.

### Eligibility Criteria

The pre-specified inclusion criteria were: (1) age 18 years or older; (2) diagnosis of COVID-19 by polymerase chain reaction (PCR) testing for SARS-CoV-2 from nasopharyngeal swabs or computed tomography scan findings compatible with the disease (bilateral multifocal ground-glass opacities ≥50%), and subsequent COVID-19 confirmation; (3) diagnosis of flu syndrome with hospitalization criteria on hospital admission, presenting with respiratory rate >24 breaths per min, saturation <93% on room air or risk factors for complications (e.g., heart disease, diabetes, systemic arterial hypertension, neoplasms, immunosuppression, pulmonary tuberculosis, obesity), followed by COVID-19 confirmation. The patients who met these criteria were considered to have moderate to severe COVID-19.

The pre-specified exclusion criteria were: (1) inability to sign the written informed consent; (2) previous admission to invasive mechanical ventilation during hospitalization; (3) creatinine ≥2 mg/dL or requiring dialysis; (4) total calcium ≥10.5 mg/dL; (5) prior vitamin D_3_ supplementation (above 1,000 IU/day); (6) pregnant or lactating women.

The criteria used for hospital discharge were: no need for supplemental oxygen in the last 48 h, absence of fever in the last 72 h (i.e., temperature ≤ 37.2°C), and oxygen saturation >93% on room air without respiratory distress (such as difficulty breathing, shortness of breath, pain, or pressure in the chest).

No patient received any SARS-COVID vaccine since at the time of data collection, there were no vaccines available for use in our population.

### Patients

Anthropometric characteristics (self-reported weight and height), acute COVID-19 symptoms, coexisting chronic diseases, patients' concomitant medications during hospitalization, oxygen supplementation requirement, imaging features, and serum 25OHD were assessed upon hospital admission. The investigation of coexisting chronic diseases was self-reported and, subsequently, all of them were checked according to the medical records of each patient, including previous medications. To provide a comprehensive demographic characterization, self-reported race data were also collected based on the following fixed categories: White, Black, Asian, and Pardo (people of mixed race/ethnicities, according to the Brazilian Institute of Geography and Statistics).

The C-reactive protein was assessed by immunoturbidimetric assay, serum 25-hydroxyvitamin was assessed by chemiluminescent immunoassay (ARCHITECT 25-OH Vitamin D 5P02, Abbott Diagnostics, Lake Forest, IL, USA), albumin was assessed by colorimetric assay, and lymphocyte count was assessed by automated assay. The analyses were performed according to the manufacturer's recommendations by the same technician in an accredited laboratory from the School of Medicine.

### Outcomes Measures

In order to provide a broader knowledge of clinical illness, hospitalized patients were categorized into groups according to oxygen therapy requirement as follows: (1) non-oxygen therapy (moderate patients not requiring oxygen therapy); (2) nasal cannula therapy (severe patients requiring nasal cannula oxygen therapy); and (3) high-flow therapy (severe patients requiring high-flow oxygen therapy) (22).

The PNI was calculated for each patient as a serum albumin (g/dL) × 10 + total lymphocyte count (per mm^3^) × 0.005. The patients were divided into 3 groups based on the PNI score: malnutrition (PNI <40), mild malnutrition (PNI = 40–45), and non-malnutrition (PNI > 45) (6). The cross-sectional analysis evaluates whether the PNI is related to COVID-19 severity which was assessed by the oxygen therapy requirement at hospital admission.

Herein, we report a *post-hoc* analysis regarding both the oxygen therapy requirement and PNI as predictors of hospital length of stay, the primary outcome. Hospital length of stay was previously defined as the total number of days that the patient remained hospitalized from study admission to hospital discharge, as published (20) and registered in ClinicalTrials.gov (NCT04449718). Likewise, the secondary outcome to understand, broadly, COVID-19-related hyperinflammation and immunomodulation regarding cytokines, chemokines, growth factor, and white blood cells have been reported (23).

### Statistical Analysis

Sample size was chosen based on the available resources and feasibility as described in detail (20, 21). Only patients with complete values for albumin and lymphocyte count were enrolled in the present study.

Generalized estimating equations (GEE) were used for testing possible differences assuming groups as fixed factors, with marginal distribution, and a first-order autoregressive correlation matrix to test the main and interaction effects. Bonferroni's adjustment was performed for multiple comparisons in GEE analyses to maintain a family-wise 2-sided significance threshold of 0.05. Proportions were compared between groups using χ^2^ and Fisher's exact tests as appropriate. In order to avoid potential confounders effects, GEE was adjusted by the center alone, and by the center and C-reactive protein. The missing data for body mass index (BMI) and 25-hydroxivitamin D were handled by GEE models with no imputation data.

The Kaplan–Meier estimate curves for oxygen therapy requirement categories (independent variables; non-oxygen therapy, nasal cannula therapy, and high-flow therapy) and PNI categories (independent variables; malnutrition, mild-malnutrition, and non-malnutrition) were compared using the log-rank, Breslow, and Tarone-Ware test for hospital length of stay (deaths were considered censored events). In order to avoid bias regarding the different weights to event in the survival curve analysis of each test, log-rank, Breslow, and Tarone-Ware were presented accordingly (24). Cox regression models were used to estimate unadjusted and adjusted hazard ratios (HR), with corresponding 2-sided, between independent variables and hospital discharge. Time from symptom onset as time-dependent covariate in the Cox regression models were used to estimate unadjusted and adjusted hazard ratios (HR), with corresponding 2-sided, between independent variables and hospital discharge. Both multivariable Cox proportional hazard models were adjusted for variables found to be associated with oxygen therapy requirement categories at cross-sectional analysis (age, sex, C-reactive protein, and diabetes) at the *P* < 0.05 level. The proportionality assumption for Cox regression models was confirmed by assessing Schoenfeld residuals. For the Cox proportional hazard models (Cox regression model and time-dependent covariate cox regression model), the outcome was labeled as hospital discharge, aiming to facilitate the interpretation of the hazard ratio for discharge. Statistical analyses were performed with the IBM-SPSS software (USA), version 20.0. The significance level was set at 2-sided *P* ≤ 0.05.

## Results

### Patients

Of the 1,600 patients evaluated for eligibility, 309 patients had complete data for PNI and were enrolled in the cross-sectional analysis; 69 in the non-oxygen therapy group, 201 in the nasal cannula therapy group, and 39 in the high-flow therapy group (Supplementary Figure 1). Of the 309 patients, 165 (53.4%) were male, 48.9% of the patients were White, the mean age was 55.8 ± 13.9 years, the mean BMI was 31.3 ± 6.6 kg/m^2^, and the median from symptom onset to study enrollment was 10 days (interquartile range, IQR, 8–13) (Table 1). Regarding PNI tool, of the 309 patients assessed at admission, 221 (71.5%) had malnutrition, 59 (19.1%) mild malnutrition, and 29 (9.4%) had non-malnutrition (Supplementary Figure 1, Table 1).

**Table 1:** Demographic and clinical characteristics according to oxygen therapy requirement at admission.

**Characteristic**	**Non-oxygen therapy group (*n* = 69)**	**Nasal cannula therapy group (*n* = 201)**	**High-flow therapy group (*n* = 39)**	**All patients (*n* = 309)**	***P***
Age, mean (SD), y	50.7 (14.1)^a^	57.8 (13.6)^b^	54.8 (12.8)^a, b^	55.8 (13.9)	**<0.01**
**Sex, no. (% within group)**					
Male	38 (55.1%)	98 (48.8%)	29 (74.4%)	165 (53.4%)	**0.01**
Female	31 (44.9%)	103 (51.2%)	10 (25.6%)	144 (46.6%)	
**Race or ethnic group, no. (% within group)**					
White	30 (43.5%)	100 (49.8%)	21 (53.8%)	151 (48.9%)	0.84^*^
Pardo	30 (43.5%)	70 (34.8%)	13 (33.3%)	113 (36.6%)	
Black	9 (13.0%)	30 (14.9%)	5 (12.8%)	44 (14.2%)	
Asian	0	1 (0.5%)	0	1 (0.3%)	
Median time from symptom onset to study enrollment (IQR), d	11.0 (8.0–14.0)	10.0 (7.0–12.2)	10.0 (7.7–12.2)	10.0 (8.0–13.0)	0.46
Body-mass index, mean (SD), kg/m^2^	30.2 (6.1) [*n* = 67]	31.7 (6.9) [*n* = 182]	31.0 (5.5) [*n* = 37]	31.3 (6.6) [*n* = 286]	0.42
25-hydroxivitamin D, mean (SD), ng/mL	20.9 (12.3) [*n* = 69]	20.9 (8.8) [*n* = 200]	23.9 (9.7) [*n* = 39]	21.3 (9.8) [*n* = 308]	0.19
C-reactive protein, mean (SD), mg/L	42.6 (44.9)^a^	78.1 (72.2)^b^	108.6 (87.4)^b^	74.0 (71.8)	**<0.001**
Lymphocyte count, mean (SD), ×103/mm3	1.4 (0.7)^a^	1.2 (1.2)^a, b^	1.0 (0.5)^b^	1.2 (1.0)	**<0.01**
Albumin, mean (SD), g/L	3.3 (0.5)^a^	3.1 (0.4)^b^	2.9 (0.3)^c^	3.1 (0.4)	**<0.01**
**Acute covid-19 symptoms, no. (% within group)**					
Cough	61 (88.4%)	171 (85.1%)	30 (76.9%)	262 (84.8%)	0.27
Fatigue	59 (85.5%)	170 (84.6%)	38 (97.4%)	267 (86.4%)	0.10
Fever	56 (81.2%)	142 (70.6%)	25 (64.1%)	223 (72.2%)	0.12
Myalgia	51 (73.9%)	129 (64.2%)	22 (56.4%)	202 (65.4%)	0.16
Joint pain	28 (40.6%)	65 (32.3%)	14 (35.9%)	107 (34.6%)	0.47
Runny nose	28 (40.6%)	87 (43.3%)	10 (25.6%)	125 (40.5%)	0.12
Diarrhea	26 (37.7%)	85 (42.3%)	15 (38.5%)	126 (40.8%)	0.77
Nasal congestion	29 (42.0%)	70 (34.8%)	11 (28.2%)	110 (35.6%)	0.33
Sore throat	32 (46.4%)	67 (33.3%)	11 (28.2%)	110 (35.6%)	0.08
**Coexisting diseases, no. (%within group)**					
Hypertension	31 (44.9%)	103 (51.2%)	20 (51.3%)	154 (49.8%)	0.66
Diabetes	14 (20.3%)	80 (39.8%)	7 (17.9%)	101 (32.7%)	**<0.01**
Cardiovascular disease	6 (8.7%)	23 (11.4%)	4 (10.3%)	33 (10.7%)	0.86
Rheumatic disease	7 (10.1%)	15 (7.5%)	6 (15.4%)	28 (9.1%)	0.26
Asthma	1 (1.4%)	15 (7.5%)	1 (2.6%)	17 (5.5%)	0.12
Chronic obstructive pulmonar disease	0	12 (6.0%)	2 (5.1%)	14 (4.5%)	0.07^*^
Chronic kidney disease	1 (1.4%)	0	0	1 (0.3%)	0.35^*^
**Concomitant medications, no. (% within group)**					
Anticoagulant	66 (95.7%)	179 (89.9%) [*n* = 199]	36 (94.7%) [*n* = 38]	281 (91.8%) [*n* = 306]	0.26
Antibiotic	61 (88.4%)	182 (91.5%) [*n* = 199]	36 (94.7%) [*n* = 38]	279 (91.2%) [*n* = 306]	0.49
Glucocorticoid	51 (73.9%)	152 (76.4%) [*n* = 199]	33 (86.8%) [*n* = 38]	236 (77.1%) [*n* = 306]	0.29
Antihypertensive	33 (47.8%)	103 (51.8%) [*n* = 199]	21 (55.3%) [*n* = 38]	157 (51.3%) [*n* = 306]	0.75
Proton pump inhibitor	41 (59.4%)	107 (53.8%) [*n* = 199]	24 (63.2%) [*n* = 38]	172 (56.2%) [*n* = 306]	0.49
Antiemetic	46 (66.7%)	115 (57.8%) [*n* = 199]	19 (50.0%) [*n* = 38]	180 (58.8%) [*n* = 306]	0.22
Analgesic	52 (75.4%)	106 (53.5%) [*n* = 198]	24 (63.2%) [*n* = 38]	182 (59.7%) [*n* = 305]	**<0.01**
Hypoglycemic	11 (15.9%)	55 (27.6%) [*n* = 199]	4 (10.5%) [*n* = 38]	70 (22.9%) [*n* = 306]	**0.02**
Hypolipidemic	11 (15.9%)	25 (12.6%) [*n* = 199]	4 (10.5%) [*n* = 38]	40 (13.1%) [*n* = 306]	0.69
Thyroid	6 (8.7%)	20 (10.1%) [*n* = 199]	2 (5.3%) [*n* = 38]	28 (9.2%) [*n* = 306]	0.64
Antiviral	2 (2.9%)	5 (2.5%) [*n* = 199]	0 [*n* = 38]	7 (2.3%) [*n* = 306]	0.86^*^
**Computed tomography findings, no. (% within group)**					
Ground-Glass opacities ≥ 50%	35 (50.7%)	119 (59.2%)	31 (79.5%)	185 (59.9%)	**0.05**
Ground-Glass opacities <50%	32 (46.4%)	73 (36.3%)	7 (17.9%)	112 (36.2%)	
PNI, mean (SD)	40.1 (6.4)^a^	37.2 (7.5)^a^	34.5 (3.7)^b^	37.5 (7.1)	**<0.001**
**PNI distribution, no/total no. (% within group)**					
Malnutrition (PNI <40)	33 (47.8%)	151 (75.1%)	37 (94.9%)	221 (71.5%)	<0.001^*^
Mild malnutrition (PNI 40–45)	20 (29.0%)	37 (18.4%)	2 (5.1%)	59 (19.1%)	
Non-malnutrition (PNI > 45)	16 (23.2%)	13 (6.5%)	0	29 (9.4%)	

*Percentages may not total 100 because of rounding. IQR denotes interquartile range, and COVID-19 coronavirus disease 2019.*

*Data were analyzed by Generalized Estimating Equations (GEE) with normal distribution and identity link function with first-order autoregressive correlation matrix. Race and ethnic group were reported by the patients. Pardo is the exact term used in Brazilian Portuguese, meaning “mixed ethnicity,” according to the Brazilian Institute of Geography and Statistics. Data were missing for 4 patients (1.3%) in the median time from symptom onset to study enrollment, and 12 patients (3.9%) in the computed tomography findings. P -value represents between-group effect adjusted by the center. Categorical variables were performed using χ^2^ test.*

* *Fisher's exact test. Different letters denote statistical significance. The bold values indicate statistical significance*.

Table 1 presents baseline demographic and clinical characteristics according to oxygen therapy requirement at admission (non-oxygen therapy, nasal cannula therapy, and high-flow therapy). All groups were similar regarding race/ethnic, median time from symptom onset to study enrollment, BMI, serum 25-hydroxivitamin D, acute COVID-19 symptoms, coexisting diseases (except for diabetes), and concomitant medications (except for analgesic and hypoglycemic). There were significant differences between groups for age, sex, C-reactive protein, lymphocyte count, albumin, diabetes, computed tomography findings, mean PNI, and PNI distribution.

### Cross-Sectional

The high-flow therapy group demonstrated significant lower mean PNI compared to non-oxygen therapy [difference, −3.1 [95% CI, −5.6, −0.7), *P* < 0.01] and nasal cannula therapy groups [difference, −2, (95% CI, −3.9, −0.2), *P* = 0.03] even after adjustment by the center and C-reactive protein (Table 1). Similar results occurred after adjustment by the center and non-adjusted models (*P* < 0.001, for both adjustment). There was a significant mean difference for PNI between non-oxygen therapy and nasal cannula therapy groups in non-adjusted models [difference, 2.9 (95% CI, 0.6, 5.1), *P* < 0.01]. No significant mean difference for PNI was observed between non-oxygen therapy and nasal cannula therapy groups adjusted by the center [difference, 1.9 (95% CI, −0.8, 4.6), *P* = 0.26] and adjusted by the center and C-reactive protein [difference, 1.1 (95% CI, −1.5, 3.7), *P* = 0.89]. The highest proportion of patients with malnutrition (94.9%) occurred in the high-flow therapy group (*P* < 0.001; Table 1).

### Primary Outcome

**Figure 1:**
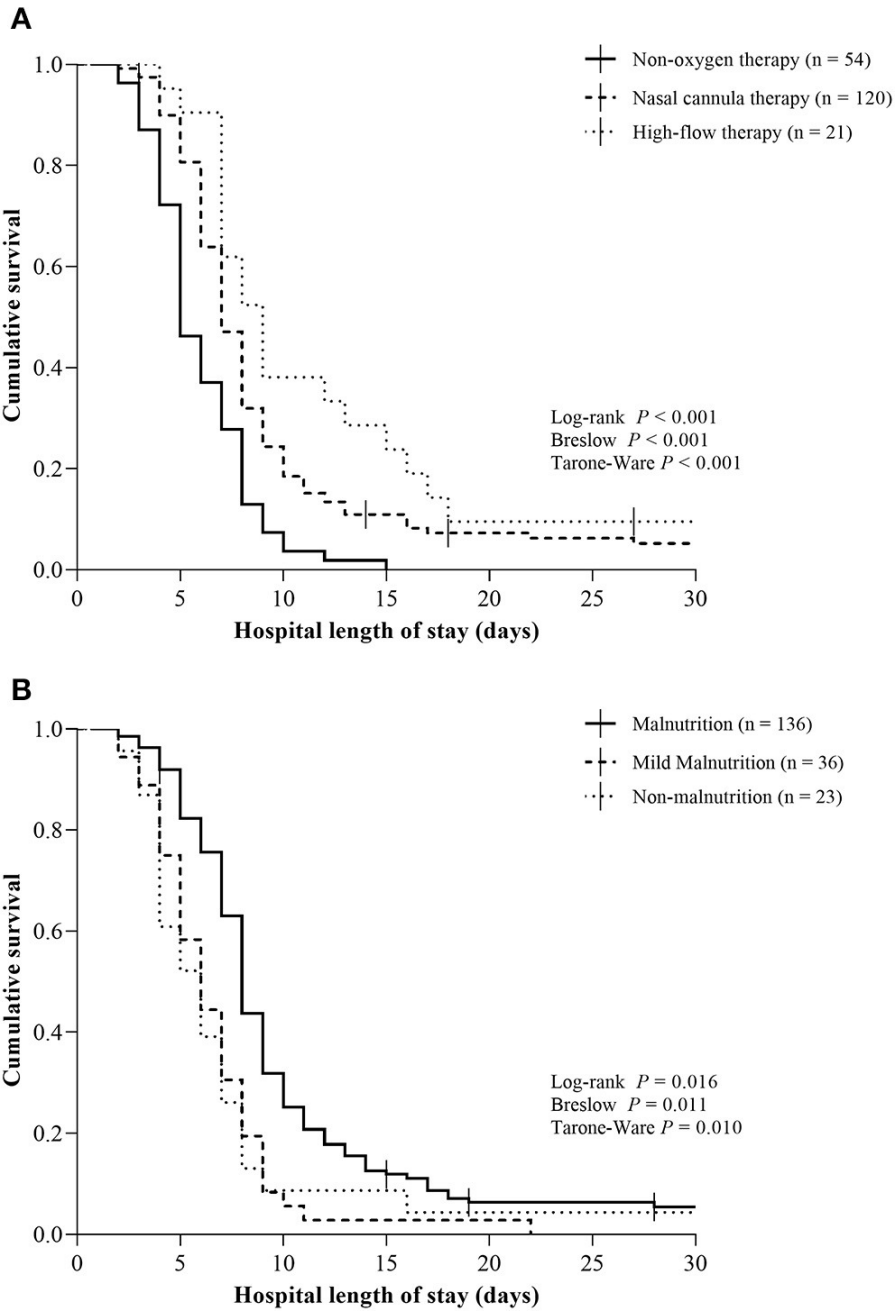
Kaplan–Meier curves for hospital length of stay according to oxygen therapy requirement and Prognostic Nutritional Index categories in patients with moderate to severe Coronavirus Disease 2019. **(A)** Non-oxygen therapy, nasal cannula therapy, and high-flow therapy; **(B)** Malnutrition (PNI <40), mild-malnutrition (PNI 40–45), and non-malnutrition (PNI > 45). Vertical bars present single censored events (deaths). Both analyses represent 195 total patients. COVID-19, Coronavirus Disease 2019.

The hospital length of stay was comparable to oxygen therapy requirement and PNI categories. There was a significant longer hospital length of stay for patients with high-flow therapy [9 days (95% CI 7.2, 10.7), *P* < 0.001] compared to nasal cannula therapy [7 days (95% CI 6.4, 7.6)] and non-oxygen therapy [4 days (95% CI 3.2, 4.8)] (Table 2, Figure 1A). Similarly, there was a significant longer hospital length of stay for patients with malnutrition [7 days (95% CI 6.6, 7.4), *P* = 0.016] compared to mild nutrition [6 days (95% CI 4.8, 7.2)] and non-malnutrition [6 days (95% CI 4.2, 7.8)] (Table 2, Figure 1B).

**Table 2:** Estimate for hospital length of stay according to oxygen therapy requirement and Prognostic Nutritional Index (PNI) categories.

**Hospital length of stay**	**No./total No.^a^**	**Estimated days, median (95% CI)**	***P* ^b^**
**Oxygen therapy requirement**			**<0.001**
Non-Oxygen therapy	54/55	4.0 (3.2, 4.8)	
Nasal cannula therapy	116/120	7.0 (6.4, 7.6)	
High-Flow therapy	20/21	9.0 (7.2, 10.7)	
**Prognostic nutritional index**			**0.016**
Malnutrition (PNI <40)	131/136	7.0 (6.6, 7.4)	
Mild malnutrition (PNI 40–45)	36/36	6.0 (4.8, 7.2)	
Non-Malnutrition (PNI > 45)	23/23	6.0 (4.2, 7.8)	

a *Number of surviving patients (death as censored events, n = 5)/total patients with available information.*

b *Log-Rank test for categorical variables. The bold values indicate statistical significance*.

The unadjusted and adjusted hazard ratio for hospital discharge according to the oxygen therapy requirement categories did show a significant lower potential to discharge for high-flow therapy [HR 0.27 (95% CI 0.16, 0.46), *P* < 0.001] and nasal cannula therapy [HR 0.38 (95% CI 0.27, 0.53), *P* < 0.001] compared to non-oxygen therapy (Table 3). The unadjusted cox regression model for hospital discharge did show a significant higher potential to discharge for patients with mild malnutrition compared to those with malnutrition [HR 1.53 (95% CI 1.05, 2.22), *P* = 0.026; Table 3]. The adjusted hazard ratio and unadjusted time-dependent covariate Cox regression model for hospital discharge according to the PNI categories did not show a significant difference (Table 3).

**Table 3:** Cox regression model and time-dependent covariate cox regression model for primary outcome according to oxygen therapy requirement and PNI categories.

		**Unadjusted**			**Adjusted^b^**		
**Hospital discharge**	**Number of events^a^**	**Hazard ratio**	**(95% CI)**	***P***	**Hazard ratio**	**(95% CI)**	***P***
**Cox regression model**
**Oxygen therapy requirement**
Non-Oxygen therapy	54	Ref.			Ref.		
Nasal cannula therapy	116	0.38	0.27, 0.53	**<0.001**	0.48	0.34, 0.68	**<0.001**
High-Flow therapy	20	0.27	0.16, 0.46	**<0.001**	0.34	0.19, 0.58	**<0.001**
**Prognostic nutritional index**
Malnutrition (PNI <40)	131	Ref.			Ref.		
Mild malnutrition (PNI 40–45)	36	1.53	1.05, 2.22	**0.026**	1.08	0.73, 1.60	0.696
Non-malnutrition (PNI > 45)	23	1.48	0.94, 2.31	0.088	1.25	0.79, 1.98	0.343
**Time-Dependent covariate Cox regression model**		**Unadjusted^c^**			**Adjusted^d^**		
**Oxygen therapy requirement**
Non-Oxygen therapy	54	Ref.			Ref.		
Nasal cannula therapy	116	0.38	0.27, 0.54	**<0.001**	0.49	0.35, 0.70	**<0.001**
High-Flow therapy	20	0.26	0.15, 0.44	**<0.001**	0.31	0.18, 0.55	**<0.001**
**Prognostic nutritional index**
Malnutrition (PNI <40)	131	Ref.			Ref.		
Mild malnutrition (PNI 40–45)	36	1.43	0.98, 2.09	0.062	0.91	0.61, 1.38	0.671
Non-Malnutrition (PNI > 45)	23	1.52	0.97, 2.38	0.070	1.21	0.76, 1.92	0.429

*PNI, Prognostic Nutritional Index.*

a *Hospital discharge refers to the number of surviving patients (death censored).*

b *Multivariable Cox regression adjusted for age, sex, C-reactive protein, and diabetes.*

c *Time from symptom onset as time-dependent covariate in the Cox Proportional-Hazard regression model.*

d *Time from symptom onset as time-dependent covariate in the Cox Proportional-Hazard regression model adjusted for age, sex, C-reactive protein, and diabetes. The bold values indicate statistical significance*.

## Discussion

To the best of our knowledge, this is the first longitudinal multicenter study assessing prospectively the PNI tool as a predictor of hospital length of stay in patients with moderate and severe COVID-19 not admitted to the ICU. Additionally, the present study is the first to assess through a multicenter cross-sectional analysis whether nutritional status, according to the PNI, is associated with the oxygen therapy requirement at admission.

According to the results, at least 90 percent of the patients with COVID-19 were malnourished at entry. These data were higher than previous evidence in patients with COVID-19 demonstrated by Doganci et al. (25) (49.7% in the lowest PNI tertile), Cinar et al. (15) (33.3% with PNI value <43.7), and Wang et al. (26) (31.7% with PNI value <43). However, these studies did not follow the recommended cut-off points (6), dividing their sample into three (15) and two (25, 26) tertiles and, therefore, making it impossible to compare the actual frequency of patients with malnutrition or mild malnutrition.

The worst nutritional status (low PNI) observed in the present study was significantly associated with increased COVID-19 severity, notably in severe patients requiring high-flow oxygen therapy compared to severe with nasal cannula oxygen therapy or moderate illness with no oxygen therapy. Our results are in agreement with individual studies (16, 26–28) and pooled effect (16) demonstrating a significantly greater disease severity at the worst PNI scores.

Patients requiring oxygen therapy, notably those undergoing high-flow therapy, as well as malnourished ones, had a longer hospital length of stay, herein. Furthermore, mild malnutrition patients presented a significant hazard ratio with greater potential to discharge compared to malnourished patients. These results were similar to those evidenced by Martinuzzi et al. (29) showing that longer hospital length of stay and longer ICU stay resulted from poor nutritional status as measured by subjective global assessment (SGA) and nutritional risk screening 2002 (NRS 2002).

Our findings reinforce the PNI as a feasible tool associated with hospital length of stay, although other factors may have contributed to the lack of statistical significance in the other models analyzed here. In line with the Vavruk et al. (1), malnourished patients admitted to the ICU evaluated using SGA had significantly higher mortality rates and longer hospital length of stay compared to well-nourished patients. Likewise, the association of oxygen therapy requirement with the reduced potential to discharge reach statistical significance in the unadjusted and adjusted Cox regression model and time-dependent covariate Cox regression model, suggesting the prognostic effect of the oxygen therapy requirement as a predictor of hospital length of stay in COVID-19 patients.

To date, the knowledge of PNI in patients with COVID-19 has been limited to cross-sectional (15, 16), small-scale cohort (14), retrospective observational (2, 15), or systematic review (16) studies. Patients with lower PNI had a higher risk of in-hospital mortality in the multivariate logistic regression of a retrospective design (2). However, one of the main disadvantages of the retrospective studies compared to prospective ones is the possible significant bias affecting the between-group comparison related to the absence of control exposure or outcome assessment, often not having adequate information about additional therapies modulating the patient's prognosis, and instead must rely on other professionals for accurate record keeping (30, 31). So, the retrospective aspect may introduce recall bias and mis-classification or information bias, resulting in inaccuracy (31).

In addition, continuing an exploratory analysis of the evidence thus far, the small-scale cohort study assessing ICU patients (14) demonstrated in obese patients a decreased PNI score at the 10th day, but not in lean patients, as well as impaired immune-inflammatory response, compromised PNI, and dysregulated metabolism to liver fat since ICU admission. Notwithstanding, the absence of a statistical between-group comparison, and the very small number of participants are important limitations that need to be considered to prevent extrapolation of these data (14).

Cinar et al. (15) retrospectively reported that PNI was independently related with in-hospital mortality in patient with higher cardiovascular risk factors and COVID-19. Despite the significant effects, patients with COVID-19 having numerous cardiovascular risk factors, such as those evaluated in this study, patients with COVID-19 carrying several cardiovascular risk factors, such as those evaluated in this study (15), *per se*, could have mitigated PNI scores due to related comorbidities or their clinical condition, such as lymphopenia and hypoalbuminemia.

Throughout hospitalization, it is well-established that the loss of lean body mass has deleterious impacts on the patient's prognosis, representing a crucial element that predisposes them to numerous adverse events in critical illnesses, including delayed recovery after discharge (32). A recent study by Di Filippo et al. (32) demonstrated that COVID-19 was associated with significant unintentional weight loss and risk of malnutrition (assessed by mini nutritional assessment screening). Of the 213 patients, 61 (29%) had a weight loss ≥5% of their initial body weight upon admission (either during hospitalization or after discharge) that was associated with greater systemic inflammation, impaired renal function, and longer disease duration compared to those who did not lose weight (32). Corroborating these data in our findings, the worst inflammatory state measured by C-reactive protein was observed in patients ongoing oxygen therapy at admission, with the highest mean values for those with high-flow (108.6 mg/L) as well as nasal cannula (78.1 mg/L) compared to non-oxygen (42.6 mg/L) therapy group, endorsing the significant hospital length of stay.

Aside from the novelty of this prospective cohort with a feasible tool, the strengths of the present study include the enrollment of hospitalized patients with moderate to severe COVID-19; patients not admitted to the ICU performing different oxygen therapies; the use of a new parameter (PNI) as a surrogate marker for oxygen requirements/severity; the assessment of nutritional status that includes realistic markers of the immune system; and the appropriate statistical adjustment regarding possible confounding variables. Nevertheless, the relatively small sample size determined according to feasibilities, resources, patients' availability, *post-hoc* analysis, and complete data is an important limitation that need to be considered. This study does not rule out the possibility that early dietary manipulations at hospital admission could be an effective strategy to improve clinical status and patients' prognosis, so further randomized clinical trials that consider this perspective would be critical.

In conclusion, among hospitalized patients with moderate to severe COVID-19, more than 90% were malnourished. Malnutrition was significantly associated with high-flow oxygen therapy requirement at hospital admission compared to nasal cannula oxygen therapy or non-oxygen therapy. Apart from oxygen therapy requirement, the worst prognostic nutritional index was associated with longer hospital length of stay, proving to be a feasible tool in the assessment of nutritional status related to the prognostic value of COVID-19 patients. Therefore, these findings do support the wide use of PNI tool in the context of hospitalizations, particularly in COVID-19, to aid multi-professional interventions ameliorating the patient's nutritional status.

## Data Availability Statement

Deidentified participant data of this study must be requested from the corresponding author upon publication (sent to rosamariarp@yahoo.com). The codebook of this study will be made available upon request by qualified clinical researchers, including a statistician, with investigator support, for specified purposes dependent on the nature of the request and the intention use of the data.

## Ethics Statement

The studies involving human participants were reviewed and approved by Ethics Committee of the Clinical Hospital of the School of Medicine of the University of São Paulo (approval numbers: 38237320.3.0000.0068) and the Ethics Committee of the Ibirapuera Field Hospital (approval numbers: 30959620.4.0000.0068). The patients/participants provided their written informed consent to participate in this study.

## Author Contributions

RP had full access to all data in the study and take responsibility for the integrity of the data and the accuracy of the data analysis. AF, BR, and RP: drafting of the manuscript. AF and BR: statistical analysis. RP: supervision. All authors designed research, conducted research, critical revision of the manuscript for important intellectual content, and have read and approved the final version of the manuscript.

## Funding

AF reports receiving grant support from the São Paulo Research Foundation (FAPESP, grant 2020/11102-2), BR reports receiving grant from Coordenação de Aperfeiçoamento de Pessoal de Nível Superior—CAPES (88887.507119/2020-00), IM reports receiving grant support from FAPESP (grant 2019/24782-4), and RP reports receiving grants support from FAPESP (grant 2020/05752-4) and from Conselho Nacional de Desenvolvimento Científico e Tecnológico (CNPq, grant 305556/2017-7).

## Conflict of Interest

The authors declare that the research was conducted in the absence of any commercial or financial relationships that could be construed as a potential conflict of interest.

## Publisher's Note

All claims expressed in this article are solely those of the authors and do not necessarily represent those of their affiliated organizations, or those of the publisher, the editors and the reviewers. Any product that may be evaluated in this article, or claim that may be made by its manufacturer, is not guaranteed or endorsed by the publisher.

## Abbreviations

PNI: prognostic nutritional index
COVID-19: coronavirus disease 2019
SARS-CoV-2: severe acute respiratory syndrome coronavirus 2
HLOS: hospital length of stay
ICU: intensive care unit
PCR: polymerase chain reaction
25OHD: 25-hydroxyvitamin D
GEE: generalized estimating equations
BMI: body mass index.
