# Supplementary material for: Prognostic Nutritional Index and Oxygen Therapy Requirement Associated With Longer Hospital Length of Stay in Patients With Moderate to Severe COVID-19: Multicenter Prospective Cohort Analyses
Source: Front Nutr. 2022 Apr 5;9:802562. doi: 10.3389/fnut.2022.802562 (PMC9037140; doi:10.3389/fnut.2022.802562)
Supplement: Supplementary file 1 [file Data_Sheet_1.PDF]

Supplementary Figure

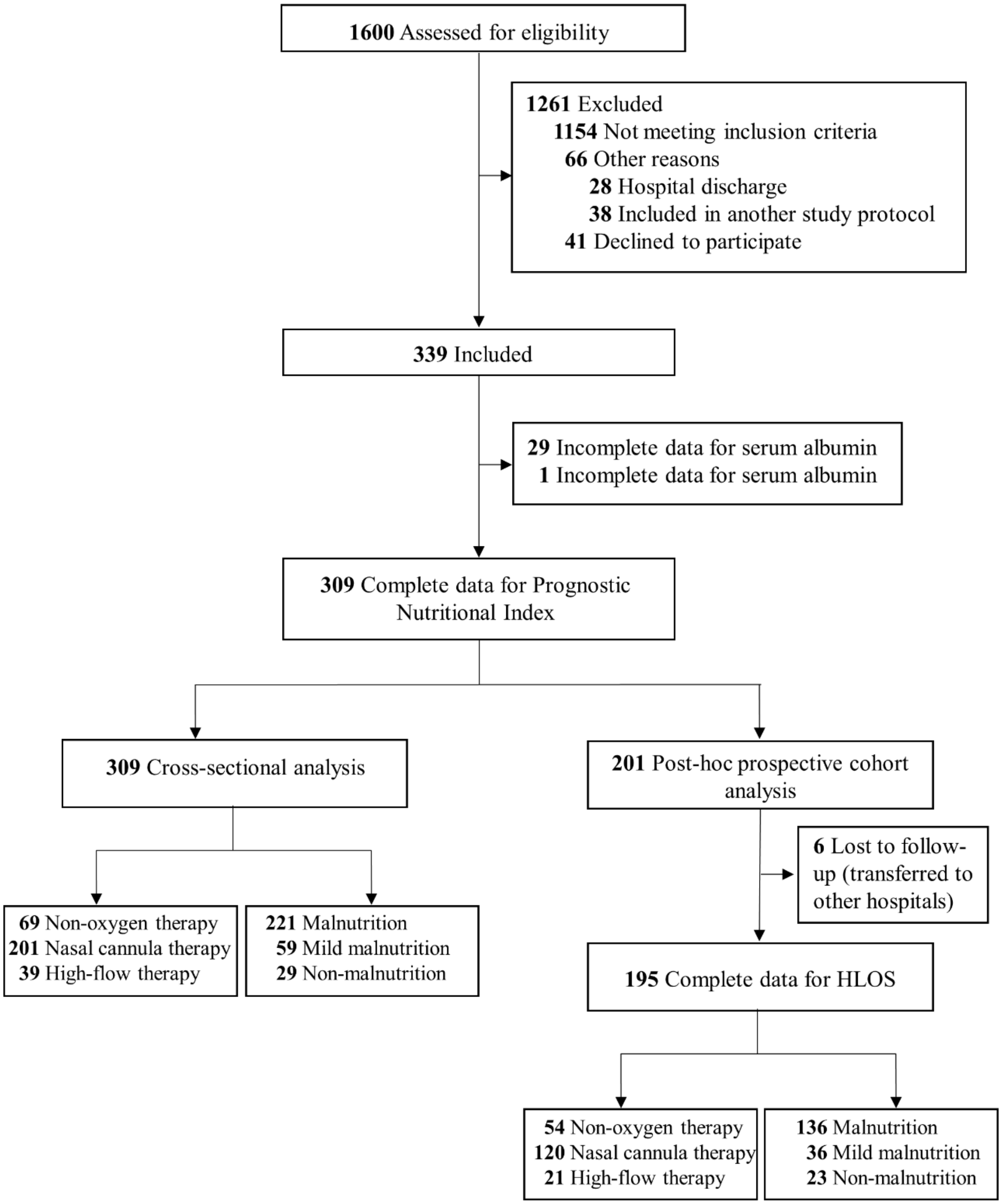

Supplementary Figure 1. Participant Flowchart. HLOS, Hospital Length of Stay
